# Prognostic Significance of CXCR4 in Colorectal Cancer: An Updated Meta-Analysis and Critical Appraisal

**DOI:** 10.3390/cancers13133284

**Published:** 2021-06-30

**Authors:** Alessandro Ottaiano, Mariachiara Santorsola, Paola Del Prete, Francesco Perri, Stefania Scala, Michele Caraglia, Guglielmo Nasti

**Affiliations:** 1 Istituto Nazionale Tumori di Napoli, IRCCS “G. Pascale”, via M. Semmola, 80131 Naples, Italy; mariachiara.santorsola@istitutotumori.na.it (M.S.); p.delprete@istitutotumori.na.it (P.D.P.); f.perri@istitutotumori.na.it (F.P.); s.scala@istitutotumori.na.it (S.S.); g.nasti@istitutotumori.na.it (G.N.); 2 BiogemScarl, Laboratory of Precision and Molecular Oncology, ContradaCamporeale, 83031 ArianoIrpino, Italy; michele.caraglia@unicampania.it; 3 Department of Precision Medicine, University of Campania “L. Vanvitelli”, Via L. De Crecchio, 7, 80138 Naples, Italy

**Keywords:** CXCR4, colorectal cancer, prognosis, overall survival

## Abstract

**Simple Summary:**

C-X-C chemokine receptor type 4 (CXCR4), a G-protein-coupled receptor, has been demonstrated to stimulate proliferation and invasiveness of many different tumors, including colorectal cancer. Through in vitro evidence, overexpression of CXCR4 has been identified as a negative prognostic factor in colorectal cancer. The identification of prognostic biomarkers can improve the prediction of disease evolution and disease characterization, and guide treatment efforts. This systematic review with a meta-analysis was conducted to pool hazard ratios from prognostic studies on CXCR4, provide an updated estimate of prognostic power of CXCR4, and analyze modalities of evaluating and reporting CXCR4 expression.

**Abstract:**

*Background:* This study was conducted to provide an updated estimate of the prognostic power of C-X-C chemokine receptor type 4 (CXCR4) in colorectal cancer (CRC), and analyze modalities of evaluating and reporting its expression. *Methods:* A systematic review with meta-analysis was performed and described according to the Preferred Reporting Items for Systematic Reviews and Meta-Analyses statement. Studies were identified through PubMed and Google Scholar. The pooled hazard ratios (HRs) for overall survival (OS) or progression-free survival (PFS) with 95% confidence interval (CI) were estimated with the random-effect model. *Results:* Sixteen studies were selected covering a period from 2005 to 2020. An immunohistochemical evaluation of CXCR4 was performed in all studies. Only in three studies assessment of mRNA through RT–PCR was correlated with prognosis; in the remaining studies, the authors identified prognostic categories based on immunohistochemical expression. In pooled analyses, significant associations were found between positive or high or strong expression of CXCR4 and T stage ≥3 (*P* = 0.0001), and positive or high or strong expression of CXCR4 and left side primary tumor localization (*P* = 0.0186). The pooled HR for OS was 2.09 (95% CI: 1.30–2.88) in favor of high CXCR4 expression; for PFS, it was 1.42 (95% CI: 1.13–1.71) in favor of high CXCR4 expression. *Conclusion:* High CXCR4 expression is clearly associated with increased risk of death and progression in CRC. However, strong methodologic heterogeneity in CXCR4 assessment hinders direct translation into clinical practice; thus, a consensus to streamline detection and scoring of CXCR4 expression in CRC is indicated.

## 1. Introduction

C-X-C chemokine receptor type 4 (CXCR4) belongs to the G-protein-coupled receptor superfamily, and it is expressed in a wide variety of cells, predominantly of hematopoietic origin. It binds to C-X-C motif chemokine ligand 1 (CXCL12), also called stromal cell-derived factor-1α (SDF-1α) and mediates a potent chemotactic stimulus [1]. In embryos, it has a major role in processes of neurogenesis, influencing the migration of neurons from neuroprogenitor cells [2]; in adults, one of the most important biological roles of the CXCR4/SDF-1α axis is the regulation of hematopoietic stem cell homing to the bone marrow [3]. However, CXCR4 has been demonstrated to stimulate proliferation and invasiveness of many different tumors including prostate [4], breast [5], lung [6,7,8], melanoma [9,10], glioblastoma [11,12], lymphoma [13,14], and colorectal cancer [15,16]. Through in vitro evidence, overexpression of CXCR4 has been identified as a negative prognostic factor in many different neoplasms [17,18,19,20].

Colorectal cancer (CRC) is the third most common cause of cancer-related deaths worldwide. Survival rates at five years strictly depend on the stage at diagnosis, varying from 90% of American Joint Committeeon Cancer 8th Edition (AJCC) stages I-II to 10% of stage IV [21]. The survival rate of Stage III patients is about 40% and it has been improved in recent years with the administration of adjuvant chemotherapy [22]. The survival of metastatic colorectal cancer (mCRC) patients significantly improved in recent years with the introduction of target-oriented drugs and a better selection of patients based on biologic/molecular characteristics (*KRAS/NRAS/BRAF* mutations, MSI, HER-2 overexpression, and other molecular markers) [23]. The identification of new prognostic cancer biomarkers is important because it can improve the prediction of disease evolution, enhance disease characterization, and guide treatment efforts. CXCR4 expression is considered a prognostic marker in CRC. However, patients′ risk stratification requires rigorous scientific validation. We previously reported that CXCR4 is able to predict progression-free (PFS) [24] and overall survival (OS) [25] in CRC. 

This study was conducted to pool hazard ratios from prognostic studies on CXCR4, provide an updated estimate of prognostic power of CXCR4, and analyze modalities of evaluating and reporting CXCR4 expression.

## 2. Materials and Methods

### 2.1. Search Strategy

This meta-analysis was performed and described according to the Preferred Reporting Items for Systematic Reviews and Meta-Analyses (PRISMA) statement [26]. Two-hundred seventy-three studies were identified through PubMed and Google Scholar searching with the following key words algorithm: “colorectal cancer” OR “colorectal tumor” OR “colorectal carcinoma” AND “CXCR4” OR “cxc chemokine receptor type 4” AND “prognosis” OR “disease free survival” OR “progression” OR “survival” (last update on 16 December 2020).

### 2.2. Study Eligibility

**Figure 1:**
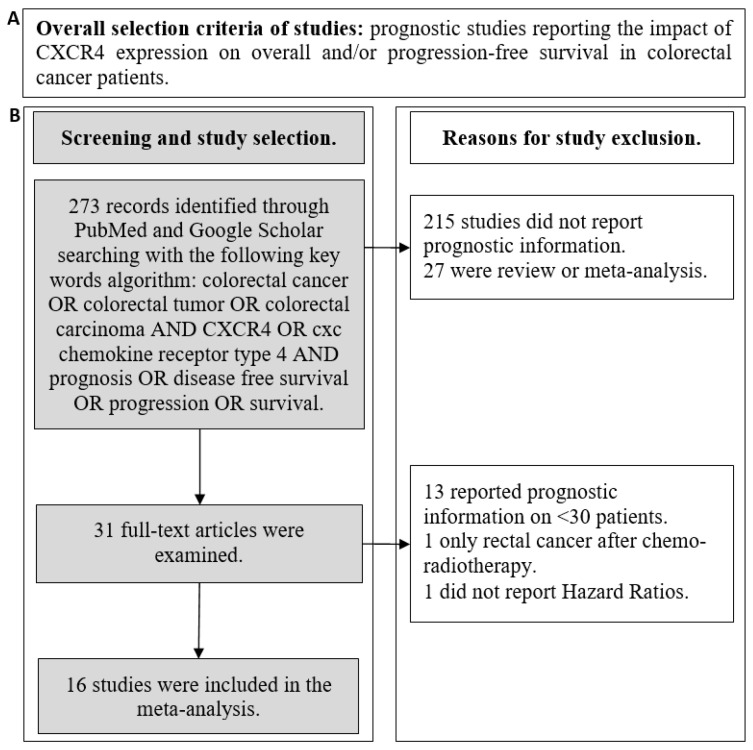
Overall selection criteria for study selection (**A**). Detailed flowchart reporting the criteria for study selection and exclusion (**B**).

A flowchart summarizing the criteria for studies selection and exclusion is reported in Figure 1. Abstracts of studies in English language that were initially identified were examined to exclude those not reporting prognostic information. Thereafter, all full texts were retrieved and analyzed. Studies were included in the final analysis if they (1) reported prognostic data (association with PFS or OS) about the expression of CXCR4 in CRC patients, (2) reported hazard ratios (HRs) with 95% confidence intervals (CIs), and, (3) had a sample size >30 patients. All the studies presented a score > 6 at the Newcastle–Ottawa Scale for methodology quality assessment [27]. Articles reporting the prognostic role of concomitant biomarker expression (i.e., CXCR4/SDF-1α, CXCR4/CD133, etc.) were excluded.

### 2.3. Data Extraction

The following data were extracted by four investigators for each publication: first author; year of publication; accrual time; number of patients; methods for CXCR4 assessment (including details on IHC scores and eventual fresh tissue evaluation); information about morphologic localization of immunohistochemical CXCR4 expression; eventual presence of ancillary studies; information on study design; association with clinico-pathological variables (age, sex, lymph-nodes involvement, stage, T, side, clinical response, and *KRAS* mutational status); information on follow-up; HRs of progression and/or death with 95% CIs. Criticisms and/or discordances were discussed between all authors to reach a consensus.

### 2.4. Hazard Ratio Interpretation

A hazard ratio of 1.0 indicates an identical risk (event probability (EP)) between high and low CXCR4-expressing groups (EP CXCR4 high/EP CXCR4 low). An HR greater than 1.0 indicates that a high CXCR4-expressing group at the numerator has an increased risk of death or progression. When a study reported an HR with low CXCR4 in the numerator (CXCR4 low vs. high), the HR and CI were recalculated (the calculated HR_CXC4 high vs. low_ was 1/HR_CXC4 low vs. high_) according to Altman et al. [28] in order to harmonize the comparison trajectory (CXCR4 high vs. CXCR4 low).

### 2.5. Statistical Analysis

The present meta-analysis was performed in order to assess the prognostic impact of CXCR4 expression in terms of OS and PFS in CRC. The secondary end-points were the analysis of the association between CXCR4 and clinico-pathologic variables, and the description of methods and scores used to assess it. Given the significant heterogeneity (see above) among the selected studies, the analysis was performed with the random-effects model. It aims to provide a more conservative estimate of the pooled HR and it is the preferred model when heterogeneity is present. Under the random-effects model, the true effects are assumed to vary between studies, and the summary effect is the weighted average of the effects reported in the different studies [29]. Meta-analysis is depicted in classical forest plots, with point estimates, 95% CIs for each HR, and a final pooled HR.

Heterogeneity was evaluated through I^2^, that is, the percentage of observed total variation across studies due to real heterogeneity rather than chance. It is calculated as I^2^ = 100% × (Q − DF)/Q, where Q is Cochran′s heterogeneity statistic and DF is the degrees of freedom. Negative values of I^2^ are set to be equal to zero so that I^2^ lies between 0% and 100%. A value of 0% indicates no observed heterogeneity, and larger values show increasing heterogeneity [30]. The risk of publication bias was also evaluated with funnel plot analysis and Egger′s test [31]. The latter is a test for the Y intercept = 0 from a linear regression of normalized effect estimate (estimate divided by its standard error) against precision (reciprocal of the standard error of the estimate). *P* < 0.005 indicates a significant publication bias.

Associations between CXCR4 expression and clinico-pathological variables were evaluated with the chi-square test.

Analyses were performed with the MedCalc Statistical Software (MedCalc^®^ Statistical Software version 19.6, MedCalc Software Ltd., Ostend, Belgium; https://www.medcalc.org (accessed on 19 December 2020).

## 3. Results

### 3.1. Study Characteristics

Sixteen studies were selected covering a period from 2005 to 2020 [24,25,32,33,34,35,36,37,38,39,40,41,42,43,44,45]. The accrual time varied from a minimum of 3 to 14 years. The number of enrolled patients ranged from 31 to 684. Only four studies reported ancillary data including evaluation of CXCR4-related biologic pathways. All studies were retrospective and had a Newcastle–Ottawa Scale score ≥ 6 (Table S1). Most articles described the prognostic power of CXCR4 in stages I-IV or stage IV disease (11/16). The reporting of association with clinico-pathological characteristics was heterogeneous (lymph-nodal status: 13/16; T status: 7/16; side: 5/16); however, very few studies reported data about association with clinical response (1/16) or *KRAS* status (2/16) (Table S2).

### 3.2. CXCR4 Expression Methodology

The methodology to assess CXCR4 expression is crucial to identifying prognostic categories and adequately interpreting results. Therefore, we performed a detailed analysis of technical modalities of CXCR4 evaluation (Table 1). Immunohistochemical evaluation of CXCR4 was performed in all studies. Evaluation of fresh tumor tissue was performed only by Kim et al. In eight studies, an mRNA assessment was added (through RT-PCR or FISH). In only three studies assessment of mRNA through RT-PCR was correlated with prognosis; in the remaining studies, the authors identified prognostic categories based on immunohistochemical expression. Ten studies differentiated nuclear versus cytoplasmic CXCR4 expression. Only one study referred to membrane CXCR4 expression. Modalities of building expression scores (number of categories, number of positive cells, and inclusion of staining intensity) were heterogeneous. A detailed description is reported in Table 1.

### 3.3. Association between CXCR4 Expression and Clinico-Pathological Characteristics of Colorectal Cancer Patients

Exploration of association between a potential biomarker in cancer and patients′ clinico-pathological characteristics is important to generate hypotheses on its biologic role, to improve disease extent prediction, and to prevent biases in subsequent prognostic analyses. Table 2 reports a detailed description of the clinico-pathological characteristics of the patients and tumors according to CXCR4 expression in the selected studies. In a pooled analysis, significant associations were found between positive or high or strong expression of CXCR4 and T stage > 3 (cancer growing outside the muscularis propria) (*P* = 0.0001), and positive or high or strong expression of CXCR4 and left-side primary tumor localization (*P* = 0.0186) (Table S3).

### 3.4. Time to Outcome According to CXCR4 Expression

The primary endpoint of this meta-analysis was to provide a pooled and updated estimate of the prognostic value of CXCR4 expression in CRC. Data regarding timeto outcome (OS and/or PFS) were extracted and are reported in Table 3. Three studies reported both OS and PFS, five reported PFS, and eight reported OS.

**Figure 2:**
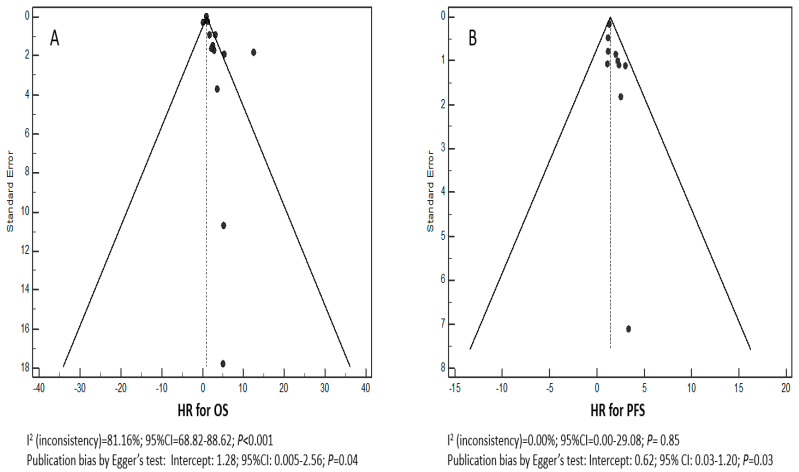
Funnel plots of selected studies for overall survival (OS) (**A**) and progression-free survival (PFS) (**B**).

**Figure 3:**
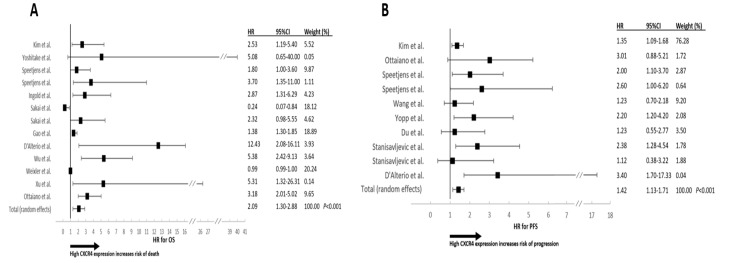
Forest plots for OS(**A**) and PFS(**B**) according to CXCR4 expression. Studies are reported by author′s first name, HRs, 95% CIs and percent of the weighted effects (see Methods). The pooled effect is reported in the last line.

Funnel plots for the HRs of OS and PFS were asymmetric (Figure 2A,B) with a significant I^2^ test for OS (*P* < 0.001). Egger′s test was significant for both OS (*P* = 0.04) and PFS (*P* = 0.03). The meta-analysis was performed with the random-effects model in order to obtain a more conservative and reliable estimate of the pooled HR, and no attempts were made to conduct a subgroup meta-analysis. A forest plot of treatment effect on OS is shown in Figure 3A. The pooled HR was 2.09 (95% CI: 1.30–2.88) in favor of high CXCR4 expression. The effect on PFS is shown in Figure 3B where the pooled HR is 1.42 (95% CI: 1.13–1.71) in favor of high CXCR4 expression.

## 4. Discussion

Identification and validation of predictive and/or prognostic biomarkers in CRC have revolutionized the management of the disease (*BRAF*, *K-*, *N-RAS*, and MSI), and others will also be used in the near future in clinical practice (HER2 and PD-1/PD-L1) [23]. Given relevant biologic reasons, CXCR4 has been explored for many years as a potential prognostic marker in CRC. Our group and others previously reported that CXCR4 expression predicted PFS and OS in CRC [24,25,32,33,34,35,36,37,38,39,40,41,42,43,44,45]. In the present study, we aimed to provide a more accurate and updated estimate of the prognostic power of CXCR4 considering some contradictory results, the maturity of available data, and the intense interest around the role of CXCR4 in modulating the metastatic behavior of CRC.

The search of articles was systematic, and the selection primarily based on a few characteristics indirectly related to the overall quality of the articles (>30 patients enrolled, HRs and CXCR4 expression methods clearly reported, and evaluation of CXCR4 before any treatment).

We found that high CXCR4 expression is clearly associated with increased risk of death and progression (HR OS: 2.09; 95% CI: 1.30–2.88 and HR PFS: 1.42; 95% CI: 1.13–1.71). However, the following major issue deserves to be evidenced and discussed: a strong methodologic heterogeneity in CXCR4 assessment was identified. Table 1 shows a wide heterogeneity in CXCR4 assessment methodology regarding techniques, scores, and categories. Some studies evaluated the membrane or nuclear expression values; however, the evaluation of the total expression value can provide an objective determination of the level of expression of CXCR4. CXCR4 nuclear localization is an atypical compartmentalization of the receptor, likely linked to its still unknown functions, and an IHC determination not sensitive enough to specifically detect the subcellular localization of a molecule, especially in paraffin-embedded tissue can be affected by artifacts and by cell space overlapping. The use of antibodies standardized for IHC studies should be recommended, and standard methods of expression evaluation scores, including the apparent subcellular localization, should be developed with an appropriate consensus between pathologists. Western blotting analysis of protein expression should be avoided because it is a qualitative method that does not distinguish the cell subtype source of the assessed protein and does not assure that the protein has been extracted by cancer cells or other tumor microenvironment cells. qRT-PCR is a quantitative technique, but it requires a strict standardization of the procedure, the selection of the tumor area by a pathologist, and the extraction of RNA from tumor tissues that are often paraffin-embedded, with the consequent decrease inthe RNA sample quality. It is likely that the best procedure would be a world-standardized IHC procedure performed with an automated test. Therefore, this methodological issue in CXCR4 assessment, along with the physiologic heterogeneity in treatments, a downsized sample size (nine studies enrolled <100 patients), and the retrospective nature of all studies are responsible for the large HR confidence intervals in some studies. Finally, the evidence of a publication bias makes these heterogeneities even more relevant. In this regard, we cannot rule out the hypothesis that selection of the studies we applied could have been influenced by this publication bias. Moreover, all selected studies were retrospective. Therefore, their nature is intrinsically biased by mostly unknown and uncontrolled clinical (patients′ location, selection, treatments, etc.) and methodological (techniques, reagents, methods, etc.) biases. Based on these considerations, our group is planning the first prospective evaluation of CXCR4 in both primary and/or metastatic tissues (resected metastases or biopsies) in order to predict the time to relapse after surgery and the response to therapy/subsequent prognosis in CRC. Assessment of CXCR4 will be performed through IHC according to a previously published homogeneous evaluation method (negative, low, high) [24,25]. The hypothesis of the study is based on the following statistical assumptions: (i) HR for high expression of CXCR4 of 2.09 (vs. negative/low), (ii) test power of 80%, (iii) alpha value of the I-type error of 5%, and (iv) median survival of 18 months (in unselected mCRC patients). The survival curves will be depicted with the Kaplan–Meier method, and the statistical significance verified with a two-tailed log-rank test. The final sample will be 200 patients.

## 5. Conclusions

For the first time, we showed a detailed and critical analysis of technical approaches applied to assess CXCR4 in CRC, finding a wide diversity in the modalities of assessment and the reporting of the receptor expression. This strong methodological heterogeneity hinders direct translation into clinical practice, suggesting that CXCR4 assessment should be revised and harmonized. Based on this, a consensus among experts to harmonize detection and scoring of CXCR4 expression in CRC should be reached; the present work may represent a critical starting point to discussions about methodological issues regarding the assessment of CXCR4 in CRC as a prognostic factor.

## Figures and Tables

**Table 1:** Description of CXCR4 assessment methodology among selected studies.

First Author	Methods	Fresh Tissue Evaluation	Detection Correlated with TTO	Differential Nuclear, Cytoplasmic, Membrane IHC Staining	IHC Distribution Correlated with Prognosis	Scores	No. of Positive Cells for Expression Evaluation	Inclusion of “Staining Intensity”
Kim J.	IHC, RT-PCR	Yes	RT-PCR	NA	NA	Low vs.High	mRNA median CXCR4 expression	No
Ottaiano A.	IHC	No	IHC	Yes	Overall expression	Neg/Low vs. High	≤50% vs.>50%	No
Yoshitake N.	IHC, Western Blot	No	IHC	Yes	Overall expression	Neg vs. Pos	No CXCR4 immunoreactivity vs others	No
Speetjens F.M.	RT-PCR	No	RT-PCR	NA	NA	Low vs. High	mRNA median CXCR4 expression	No
Speetjens F.M.	IHC	No	IHC	Yes	Nuclear expression	Weak vs. Strong	Examples are included in the work	Yes
Ingold B.	IHC	No	IHC	No	Overall expression	0, 1+, 2+, 3+	NA	Yes Absent, faint cytoplasmic, moderate cytoplasmic and slight membranous, strong cytoplasmic and strong membranous
Wang S.C.	IHC, RT-PCR	No	IHC	Yes	Nuclear expression	0, 1+, 2+, 3+	0; <30%; 30–50%; >50%	Yes Weak, medium, strong, very strong
Yopp A.C.	IHC	No	IHC	Yes	Overall expression	Neg vs. Pos	≤10% vs.>10%	No
Sakai N.	IHC, fluorescence microscopy	No	IHC	Cyto	Cytoplasm	Low vs. High	NA	Yes Relative to the staining intensity of hepatocytes
Sakai N.	IHC, fluorescence microscopy	No	IHC	Nucleus	Nuclear	Neg vs. Pos	NA	Yes Relative to the staining intensity of hepatocytes
Du C.	IHC	No	IHC	Not Specified	Overall expression	0, 1+, 2+: Low3+: High	<1%; 1–50% (1+ and 2+); >50%	Yes Negative, weak, strong
Gao Y.	IHC, RT-PCR	No	IHC	No	Overall expression	Sporadic, focal, diffuse	<10%, ≥11<50%, ≥50%	Yes Negative, weak, moderate, strong
Stanisavljevic L. (cohort 1)	IHC, ISH	No	IHC	Yes	Nuclear expression	Low vs. High	0–20%, >20%	No
Stanisavljevic L. (cohort 2)	IHC	No	IHC	Yes	Nuclear expression	Low vs. High	0–20%, >20%	No
D′Alterio C.	IHC, RT-PCR	No	IHC	Yes	Overall expression	Negative/Low vs. High	0–50%, >50%	No
Wu W.	IHC, RT-PCR	No	RT-PCR	NA	NA	Low vs. High	mRNA median CXCR4 expression	No
Weixler B.	IHC	No	IHC	No	Overall expression	Histoscores (a continuous variable)	(% of positive cells)x (staining intensity)	Yes Negative, 0; weak, 1; moderate, 2; strong, 3
Xu C.	IHC, RT-PCR	No	IHC	No	Membrane	0, 1, 2, 3, 4	0%, 1–25%, 26–50%, 51–75%, >75%	Yes No staining, weak, moderate, strong
Ottaiano A.	IHC, RT-PCR	No	IHC	Yes	Overall expression	Neg/Low vs. High	≥0≤50%, >50%	No

IHC: Immunohistochemistry; ISH: In situ hybridization; mRNA: messenger ribonucleic acid; NA: not applicable; Neg: negative; Pos: positive; RT-PCR: reverse transcriptase-polymerase chain reaction; TTO: time to outcome.

**Table 2:** Clinico-pathological characteristics according to CXCR4 expression.

Author	Year	CXCR4 Scores	Age		Sex		T		Side	Lymph nodes		
			Young	Old	Male	Female	≤2	≥3	Left	Right	Involved	Not Involved
Kim J.	2005	Low 44			23	21	18	9	-	-	8	36
		High 48			22	26	11	19	-	-	8	40
Ottaiano A.	2006	Neg 16	<70:10	≥70:6	6	10	5	11			0	15
		Low 25	<70:13	≥70:12	15	10	13	12			8	18
		High 31	<70:21	≥70:10	17	14	11	20			7	24
Yoshitake N.	2008	Negative 13	-	-	10	3	-	-	-	-	7	6
		Positive 47	-	-	31	16	-	-	-	-	38	9
Speetjens F.M.	2009	Low 35	<68.5:20	>68.5:15	16	19	-	-	17	18	12	23
		High 35	<68.5:15	>68.5:20	19	16	-	-	17	18	11	24
Speetjens F.M.	2009	Strong 43	<69.7:21	>69.7:22	21	22	-	-	22	21	15	28
		Weak 15	<69.7:8	>69.7:7	7	8	-	-	5	10	4	11
Ingold B.	2009	Negative 267	≤65:115	>65:152	145	122	46	206	-	-	133	119
		Positive 135	≤65:51	>65:84	69	66	23	108	-	-	72	59
Wang SC.	2010	Negative 245	-	-	180	65	138	107	186	59	142	141
		Positive 143	-	-	89	54	47	96	99	44	101	158
Yopp A.C.	2012	Negative 28	≤60:11	>60:17	13	15	-	-	-	-	-	-
		Positive 47	≤60:21	>60:26	38	9	-	-	-	-	-	-
Sakai N.	2012	Low 56 (cytoplasm)	<60:26	≥60:30	36	20	-	-	-	-	-	-
		High 36 (cytoplasm)	<60:10	≥60:26	22	14	-	-	-	-	-	-
Du C.	2014	Low 89	<65:39	≥65:50	51	38	16	73	47	42	6	83
		High 56	<65:19	≥65:37	33	23	9	47	29	27	4	52
Gao Y.	2014	Negative 512	<55:243	≥55:269	292	220	-	-	-	-	201	311
		Positive 208	<55:89	≥55:119	120	88	-	-	-	-	138	70
Stanisavljevic L.	2015	Low 78	-	-	45	33	3	75	-	-	-	-
		High 186	-	-	93	93	10	176	-	-	-	-
Stanisavljevic L.	2015	Low 35	-	-	20	15	6	29	-	-	-	-
		High 190	-	-	110	80	28	162	-	-	-	-
D′Alterio C.	2016	Negative/Low 10	-	-	-	-	-	-	-	-	-	-
		High 21	-	-	-	-	-	-	-	-	-	-
Wu W.	2016	Low 40	-	-	-	-	-	-	-	-	-	-
		High 40	-	-	-	-	-	-	-	-	-	-
Weixler B.	2017	Low 289	-	-	145	144	51	230	205	82	142	141
		High 267	-	-	117	150	60	204	190	77	101	158
Xu C.	2018	Low 26	<60:21	≥60:5	16	10	4	22	-	-	1	25
		High 22	<60:20	≥60:4	18	4	5	17	-	-	5	17
Ottaiano A.	2020	Negative/Low 26	≤65:12	>65:14	17	9	-	-	19	7	-	-
		High 52	≤65:20	>65:32	27	25	-	-	26	26	-	-

**Table 3:** Follow-up, time to outcome, and hazard ratios of progression and/or death in selected studies.

Author	Year	Median Follow-Up (Months)	Median PFS (Months)	HR	CI	*P*	Median OS (Months)	HR	CI	*P*
Kim J.	2005	28	NR	1.35	1.09–1.68	0.0065	High 9; Low 23	2.53	1.19–5.40	0.016
Ottaiano A.	2006	23	NR	3.01	0.88–5.21	0.0991	NR			
Yoshitake N.	2008						NR	5.08	0.65–40.00	0.123
Speetjens F.M.	2009	NR	NR	2	1.1–3.7	0.03	NR	1.8	1–3.6	0.07
Speetjens F.M.	2009	NR	NR	2.6	1–6.2	0.04	NR	3.7	1.35–11	0.02
Ingold B.	2009	32					NR	2.87	1.31–6.29	0.009
Wang SC.	2010	61	5 years DFS rate: High 70%; Low 55% (Nuc CXCR4)	1.23	0.7–2.18	0.458				
Yopp A.C.	2012	68	Pos. 15 vs Neg. 73	2.2	1.2–4.2	0.012				
Sakai N.*	2012	38					3 years OS rate: High 67%; Low 78%	Cyto: 0.43	Cyto: 0.18–1.02	Cyto: 0.056
Sakai N.*	2012	38					3 years OS rate: Pos 93%; Neg 67%	Nuc: 4.05	Nuc: 1.19–13.8	Nuc: 0.025
Du C.*	2014	68.5	5 years DFS rate: High 76.8%; Low 84.3%	0.81	0.36–1.8	0.618				
Gao Y.	2014						NR	1.3	1.38–1.85	0.001
Stanisavljevic L.*	2015	Min from 3–5 years	5 years DFS rate: High 65%; Low 85%	0.42	0.22–0.78	0.006				
Stanisavljevic L.*	2015	Min from 3–5 years	High 82%; Low 89%	0.89	0.31–2.61	0.838				
D′Alterio C.*	2016	28	High 14 vs. Neg/Low 46	3.405	1.70–17.33	0.004	High 28; Neg/Low 46	0.079	0.062–0.480	0.0008
Wu W.	2016	Max 60					NR	5.38	2.42–9.13	0.002
Weixler B.	2017	NR					5 years OS rate: High 48%; Low 48%	0.99	0.99–1.0	0.322
Xu C.*	2018	NR					High 51; Low 54	0.188	0.03–0.75	0.020
Ottaiano A.	2020	53					High 19; Neg/Low 31	3.18	2.01–5.02	0.0312

* HR was transformed in forest plot (see Methods).CI: confidence interval; Cyto: cytoplasmic; DFS: disease-free survival; HR: hazard ratio; Neg: negative; NR: not reported; Nuc: nuclear; OS: overall survival.

## Data Availability

The data presented in this study are available on request from the corresponding author.

## Supplementary Materials

The following are available online at https://www.mdpi.com/article/10.3390/cancers13133284/s1. Table S1: Study characteristics I, Table S2: Study characteristics II, Table S3: Pooled analysis of CXCR4 expression according to clinico-pathological characteristics.
