# Supplementary material for: Prognostic Significance of CXCR4 in Colorectal Cancer: An Updated Meta-Analysis and Critical Appraisal
Source: Cancers (Basel). 2021 Jun 30;13(13):3284. doi: 10.3390/cancers13133284 (PMC8269109; doi:10.3390/cancers13133284)
Supplement: Supplementary file 1 [file cancers-13-03284-s001.zip › cancers-1281939-supplementary.pdf]

Table S1. Study characteristics I.

First Author	Year	Accrual time	No. of pts	Associated ancillary study	Study design	Newcastle-Ottawa Scale score
Kim J.	2005	1996 - 2002	125	No	R	7
Ottaiano A.	2006	2003 - 2004	72	Yes	R	7
Yoshitake N.	2008	1990 - 2003	60	Yes	R	6
Speetjens F.M.	2009	1990 - 2001	70[RT-PCR]	No	R	7
Speetjens F.M.	2009	1990 - 2001	58[IHC]	No	R	7
Ingold B.	2009	1995 - 2007	402	No	R	6
Wang SC.	2010	2001 - 2004	388	Yes	R	7
Yopp A.C.	2012	2002 - 2004	75	No	R	6
Sakai N.	2012	1999 - 2007	92[Cytoplasm]	No	R	7
Sakai N.	2012	1999 - 2007	92[Nuclear]	No	R	7
Du C.	2014	2000 - 2007	145	No	R	7
Gao Y.	2014	2003 - 2008	720	No	R	7
Stanisavljevic L.	2015	1993 - 1996	264[Cohort 1]	No	R	7
Stanisavljevic L.	2015	2007 - 2011	225[Cohort 2]	No	R	7
D'Alterio C.	2016	2007 - 2009	31	No	R	6
Wu W.	2016	2007 - 2009	80	Yes	R	7
Weixler B.	2017	Not reported	684	No	R	7
Xu C.	2018	2012 - 2017	48	No	R	7
Ottaiano A.	2020	2004 - 2010	78	No	R	7

IHC:ImmunoHistoChemistry; pts: patients; R: Retrospective; RT-PCR: Reverse Transcriptase-Polymerase Chain Reaction.

Table S2. Study characteristics II.

Fist Author	Year	Stage*	Association with lymph-nodal status	Association with T status	Association with side	Association with clinical response	Association with KRAS status
Kim J.	2005	I to IV	Yes	Yes	No	No	No
Ottaiano A.	2006	I-III	Yes	Yes	No	NA	No
Yoshitake N.	2008	I to IV	Yes	No	No	No	No
Speetjens FM.	2009	I-III	Yes	No	Yes	NA	No
Ingold B.	2009	I-IV	Yes	Yes	No	No	No
Wang SC.	2010	I-IV	Yes	No	Yes	No	No
Yopp A.C.	2012	IV	No	No	No	No	No
Sakai N.	2012	IV	Yes	No	No	No	No
Du C.	2014	I-III	Yes	Yes	Yes	No	No
Gao Y.	2014	I-IV	Yes	No	No	NA	No
Stanisavljevic L.	2015	I-III	Yes	Yes	No	No	No
D'Alterio C.	2016	IV	No	No	No	NA	Yes
Wu W.	2016	IV	Yes	No	No	No	No
Weixler B.	2017	I-III	Yes	Yes	Yes	NA	No
Xu C.	2018	I-IV	Yes	Yes	No	No	No
Ottaiano A.	2020	IV	No	No	Yes	Yes	Yes

*According to AJCC (American Joint Committee on Cancer 8th Edition)

Table S3. Pooled analysis of CXCR4 expression according to clinico-pathological characteristics.

**CXCR4 expression**	**Age no. (%)**		***P***	**Gender no. (%)**		***P***	**T no. (%)**		***P***	**Side no. (%)**		***P***	**Lymphnodes no. (%)**		***P***
	**Young**	**Old**		**Male**	**Female**		**≤2**	**≥3**		**Left**	**Right**		**Involved**	**Not involved**	
Negative/Low/ Weak	518 (64.4)	577 (60.3)		1037 (55.1)	777 (52.6)		300 (59.5)	666 (44.0)		293 (43.3)	218 (50.6)		664 (57.0)	929 (59.3)	
Positive/High/ Strong	287 (35.6)	380 (39.7)	*0.0805*	846 (44.9)	700 (47.4)	*0.1548*	204 (40.5)	849 (56.0)	*0.0001*	383 (56.7)	213 (49.4)	*0.0186*	500 (43.0)	639 (40.7)	*0.2483*
